# Septic Embolic Stroke Followed by Hemorrhage and Brain Abscess in a Patient with Systemic Infections: A Case Report and Literature Review

**DOI:** 10.1155/2018/4602352

**Published:** 2018-05-03

**Authors:** Jama A. Mohamud, Jingtao Wu, Ye Jing, Yu Wang

**Affiliations:** ^1^Department of Medical Imaging, Subei People's Hospital of Jiangsu Province, Medical College of Yangzhou University, Yangzhou, China; ^2^Department of Medical Imaging, Subei People's Hospital of Jiangsu Province, Yangzhou University, Yangzhou, China; ^3^Department of Neurology, Subei People's Hospital of Jiangsu Province, Dalian Medical University, Dalian, Liaoning Province, China

## Abstract

We report a case of 50-year-old man with a severe acute ischemic stroke followed by intracerebral hemorrhage and brain abscess due to systemic infection. His initial intracranial radiographic findings were normal but three days later MRI scan of the brain revealed well-defined rounded cystic lesion on the T2-weighted and T1-weighted images in the right basal ganglia; the lesion presented an area of diffusion restriction on DWI; lately the lesion was confirmed to be an early stage of cerebral abscess. A week later the patient was noted to have worsening neurological status and left extremity weakness, and emergency brain CT scan revealed massive intracerebral hemorrhage in the right occipital lobe; he underwent intracranial hematoma evacuation surgery. The hematoma was removed successfully, and the systemic infections were treated with antibiotics.

## 1. Introduction

Ischemic stroke is life-threatening and severe medical condition requiring urgent and comprehensive evaluation. Systemic infections can increase the risk of ischemic stroke and may worsen ischemic stroke (IS) prognosis. Acute infections of different types constitute a risk factor for IS, particularly within one week of the event [1, 2]. We report here computed tomography (CT) and the magnetic resonance imaging (MRI) findings and serial image changes in a case of an acute ischemic stroke followed by intracerebral hemorrhage and brain abscess caused by systemic infection; to the best of our knowledge, this has not been reported in the literature before.

## 2. Case Report

**Figure 1:**
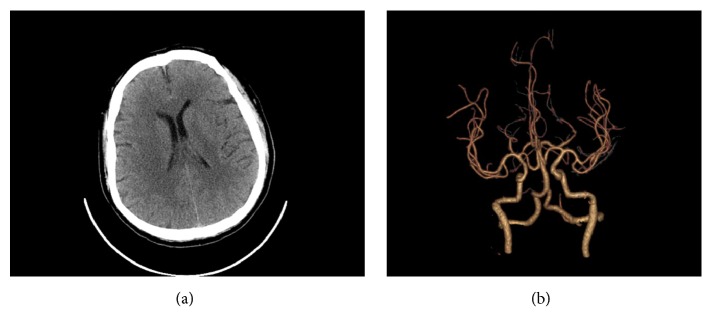
Initially, (a) a noncontrast computed tomography (CT) and (b) a computed tomography angiography (CTA) of the head performed on at the time of the admission showed no abnormalities and vascular malformations.

A 50-year-old man with a past history of hypertension and diabetes was referred to our hospital after the onset of left-sided numbness and weakness for 5 hours. The patient had not any other past medical history other than hypertension and diabetes, and there was no family history of similar diseases. A preoperative computed tomography (CT) of the head and CT angiogram of the brain (CTA) scan on admission showed no abnormalities and vascular malformations (Figures 1(a) and 1(b)). He was transferred to the intensive care unit (ICU) for further investigations and treatment. He was given an intensive care such as oxygen therapy and lipid-lowering.

Initial examination at the time of admission showed a temperature of 39°C, blood pressure of 140/92 mmHg, and a regular heart rate of 78/minute. His Glasgow coma scale (GCS) score was 7, pupils were equal and reacting, and there was grade 3 power in the left limb and grade 5 in the right limb. Further laboratory examination showed a leukocyte count of 16.5 × 109/L, a hemoglobin level of 129 g/L, neutrophil count of 83.4%, blood gas of pH 7.463, PO2 88.4, PCO2 26.8, and glucose in urine 4+; his hypersensitive c-reactive protein was 497.4 mg/L (normal < 3 mg/L). Echocardiography was performed showing a reduced compliance of left ventricle; the E-F slope of anterior mitral valve leaflet was decreased; otherwise, the size, structure, and movement of the heart were normal.

**Figure 2:**
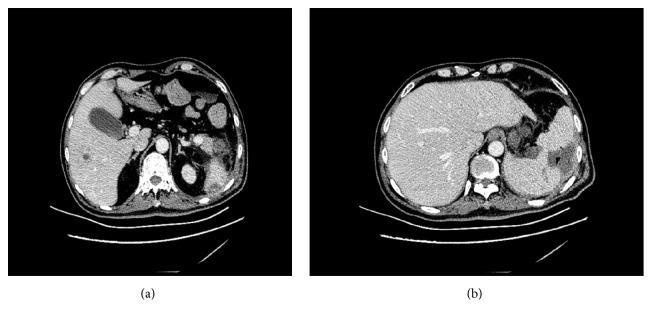
A contrast-enhanced CT scan of abdomen showed (a) rounded hypodense lesion with central low attenuation in the right lobe of the liver (suggestive of liver abscess) and (b) hypodense splenic air-filled lesion with an irregular wall (suggestive of perisplenic infection).

The patient was diagnosed with liver abscess, perisplenic and intracranial infection, and septicemia; a contrast-enhanced CT scan of the abdomen showed rounded hypodense lesion with central low attenuation in the right lobe of the liver and a hypodense splenic air-filled lesion with an irregular wall (Figure 2). Ultrasonically guided percutaneous drainage of the splenic cyst fluid was performed; approximately 65 ml of light yellow, murky fluid was removed. The blood cultures and splenic cyst drainage were positive with Klebsiella pneumoniae which were the primary source of septicemia.

**Figure 3:**
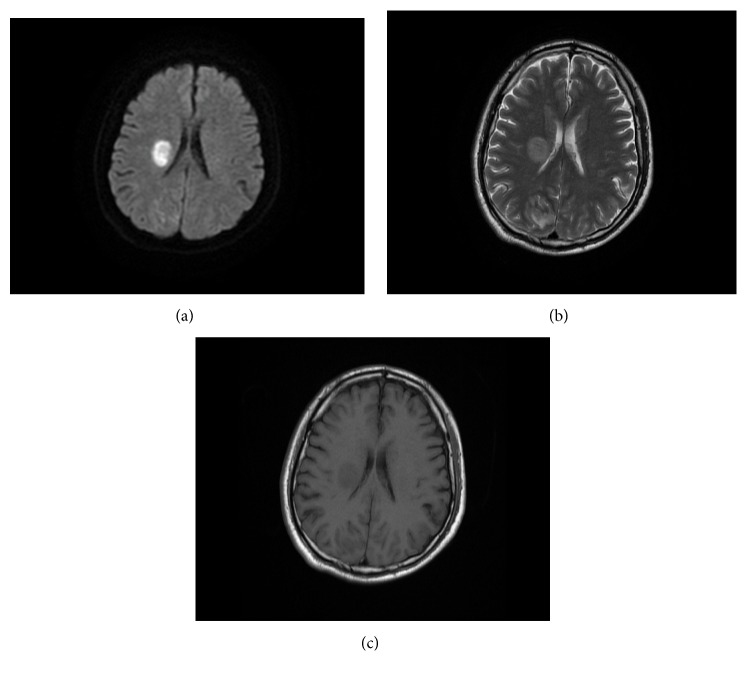
Brain MRI performed on the third day revealed (a) high signal intensity on DW image and (b, c) well-defined rounded cystic lesion on the T2-weighted and T1-weighted images involving in the right corona radiata and basal ganglia, compressing the right lateral ventricle with midline shift to left, suggestive of an early stage of brain abscess.

**Figure 4:**
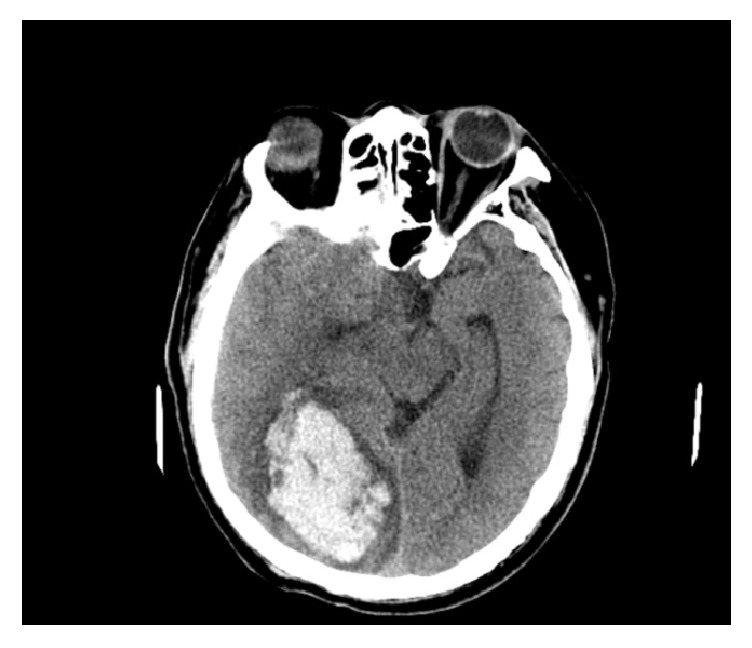
Noncontrast head CT performed on the 7th day demonstrates an acute hemorrhage in the right occipital lobe, with surrounding oedema and compressing the right lateral ventricle with midline shift to left.

**Figure 5:**
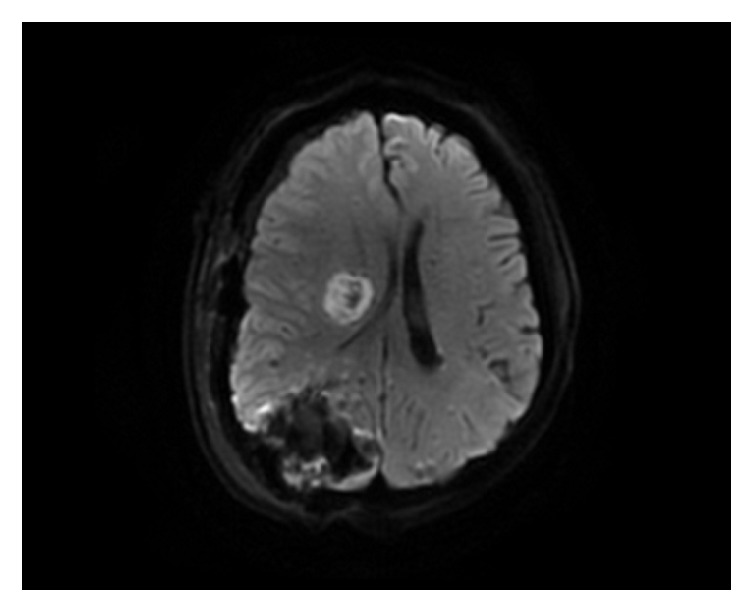
A contrast-enhanced MR imaging of brain performed three days after operation showed irregular mixed signal intensity on DWI with enhancement of its wall in the right occipital lobe and hyperintense lesion with small hypointense areas within in the basal ganglia region.

On the third day in the hospital, magnetic resonance imaging (MRI) of the head showed high signal intensity on DW image and well-defined rounded cystic lesion on the T2-weighted and T1-weighted images involving in the right corona radiata and basal ganglia (Figure 3), although the lesion looks like that of an acute stroke but lately was confirmed to be early stage of cerebral abscess. On the seventh day, the patient was noted to have worsening neurological status and left extremity weakness and a repeat computed tomography (CT) scan revealed a right occipital lobe hematoma with blood loss of 40 ml (Figure 4). He was immediately admitted to the surgical ward to undergo an urgent intracranial hematoma evacuation surgery. During the first three postoperative days the patient was delirious; further MRI of the brain three days after operation showed multiple hemorrhagic lesions involving both cerebral hemispheres and cerebellum and a cystic lesion in the basal ganglia region (Figure 5).

**Figure 6:**
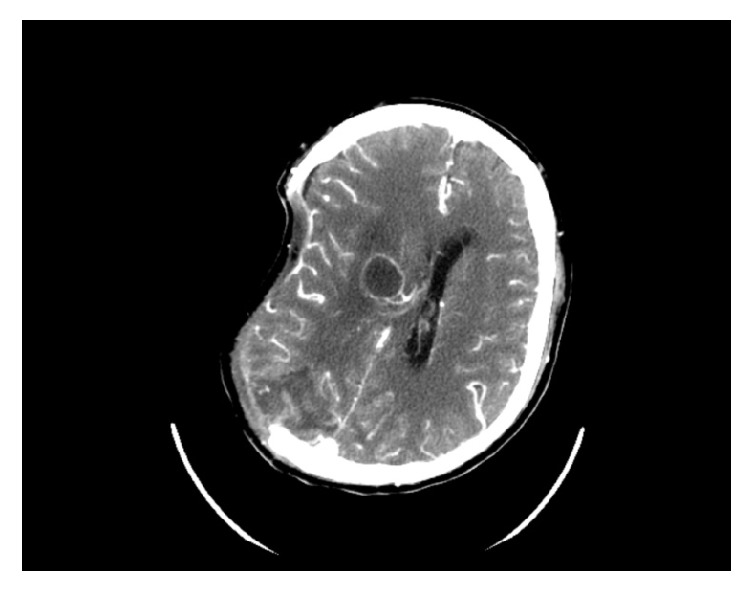
Contrast-enhanced CT of head 30 days after the initial ischemia showed a ring-enhancing cystic lesion in the right basal ganglia with perilesional oedema, which compressed right lateral ventricle with midline shift towards left suggestive of abscess.

On hospital day 34, the patient developed high-grade fevers accompanied by worsening neurological status. Contrast-enhanced CT of the head showed a well-defined lesion, with thin, ring-like enhancement pattern in the right basal ganglia with perilesional edema, which compressed right lateral ventricle with midline shift towards left and was given the possibility of brain abscess (Figure 6). The patient was treated conservatively with supportive care; he was given an antiplatelet and anti-infective therapy; the antibiotic drugs that he was given were included imipenem, linezolid, and vancomycin. He was discharged to home after two months in stable condition and slow improvement in his mental status but he still had some weakness on the left side of his body.

## 3. Discussion

Stroke has been defined as one of the most complicated pathologies; patients with stroke are at risk of developing a wide range of complications to their stroke, in particularly, stroke-associated infections increase the mortality and morbidity rates [1]. Acute ischemic and hemorrhagic stroke followed by brain abscess during the same hospitalization period is a rare entity and association of these three life-threatening conditions may cause catastrophic result; it appears possible that acute infections of bacterial etiology, usually hepatic and of bacterial origin particularly in one week, may increase the risk factor for cerebral infarction in all age groups.

Septic embolic stroke usually results from vascular occlusion and corresponding degrees of ischemia and infarction, depending on vessel size, location, and collateral blood flow [3]. Cerebral arterial occlusion resulting in either infarction or transient ischemic attack accounts for 40–50% of central nervous system (CNS) complications of infective endocarditis [4]. The septic emboli are challenging because they involve three important cerebrovascular conditions: (a) cerebrovascular occlusions, (b) intracerebral abscess, and (c) arterial mycotic aneurysms. The main risk of neurologic complications is the absence of appropriate antibiotic therapy. It is important to note that most neurologic complications are already evident at the time of hospitalization or develop within a few days [5, 6].

The clinical course in the present case revealed unusual features and complex clinical manifestation. On his initial examinations, he was diagnosed with septicemia, liver abscesses, and perisplenic and intracranial infection indicating the presence of systemic infection and bacterial seeding in the body.* Klebsiella pneumoniae* was isolated from both the blood culture and splenic cyst drainage. Hence we hypothesize that hematogenous spread from the* K*.* pneumoniae* led to bacterial seeding of the ICH and the formation of the brain abscess, in the setting of impaired host defense mechanisms. The* k*.* pneumonia* bacteria entered the bloodstream and spread to the brain and thereby led to arterial thrombosis and the destruction of the vascular wall that resulted in right cerebral ischemia and the ICH; during the operation, we had seen multiple ruptured arterioles under the hematoma wall which was responsible for the bleeding vessels in the brain. This event illustrates that septic embolism can be considered as a cause of ischemic stroke and brain abscess in patients with a hepatic abscess.

Our patient was diabetic and hypertensive and did not have any other risk factors. Initial head CT scans done on admission time were normal. Three days later he developed acute ischemic stroke and brain abscess at different locations after an infectious complication. Cerebral abscesses are difficult to diagnose and they often mimic cerebral infarcts. In the earliest stages of cerebral abscess, a CT scan of the brain may be negative or it may show subtle nonspecific findings. However, MRI findings with a diffusion protocol are more helpful in differentiating cerebral stroke and abscess. Previous studies have reported that the formation of the cerebral abscess after a stroke is a rare event and serious condition that requires immediate recognition and treatment. However the need for having a high index of suspicion for septic stroke and cerebral abscess formation is necessary for early management, diagnosis, initiation of effective treatment, and prevention of significant morbidity and death [7, 8].

## 4. Conclusion

Intracranial hemorrhage after ischemic infarct due to septic emboli is a rare mechanism.* K*.* pneumoniae* is able to progress into severe bacterial infections leading to bloodstream infections and may lead to subsequent spread to brain parenchyma even in the absence of cyanotic heart disease. However, the recognition of infectious diseases and their risk factors for septic embolic stroke could be important for stroke prevention and may help to understand epidemiologic features of septic stroke.
